# “Multiple Mode Procedures” of Ultra-Pulse Fractional CO_2_ Laser: A Novel Treatment Modality of Facial Atrophic Acne Scars

**DOI:** 10.3390/jcm12134388

**Published:** 2023-06-29

**Authors:** Zhonglan Pan, Yanqiu Tang, Hui Hua, Zuoqiong Hou, Bingrong Zhou

**Affiliations:** 1 Nanjing Yijia Medical Aesthetic Clinic, Nanjing 210029, China; pzl890622@163.com; 2 Department of Dermatology, The First Affiliated Hospital of Nanjing Medical University, Nanjing 210029, China; tyq1026@163.com; 3 Department of Dermatology, Nantong Third People’s Hospital Affiliated to Nantong University, Nantong 226000, China; huahui@ntu.edu.cn; 4 Department of Burn and Plastic Surgery, The First Affiliated Hospital of Nanjing Medical University, Nanjing 210029, China

**Keywords:** ultra-pulse CO_2_ laser, multiple mode procedures, acne atrophic scar

## Abstract

Background and aim: Fractional CO_2_ laser is therapeutic for acne atrophic scar, but its effect usually is limited after multiple sessions, with occasional adverse reactions. This study aimed to evaluate the efficacy and safety of a new modality combining ultra-pulse CO_2_ laser and fractional CO_2_ laser (multiple mode procedures [MMP]) in the treatment of acne atrophic scars. Method: From December 2017 to January 2023, a total of 103 patients with facial acne atrophic scars treated with MMP technique were retrospectively analyzed. MMP was performed for 1–4 sessions with an interval of approximately three months. Based on photographs taken before and after treatment, overall atrophic scar improvement was assessed according to the ECCA grading scale, the modified Manchester Scar Scale and the 4-point Global Assessment Scale (GAS). The safety was evaluated by the degree of pain during treatment and postoperative adverse reactions. Results: All the 103 patients received treatment and completed follow-up, with an average of two sessions. The mean ECCA score decreased from 162.7 to 93.1 with statistically significant difference (*p* < 0.001). The mean GAS score increased by an average of 2.3 ± 0.9. The GAS improvement more evident for “boxcar” atrophic scars (2.7 ± 0.8) than for “rolling” (2.3 ± 0.8) and “icepick” scars (1.7 ± 0.8) (*p* < 0.001). The average improvement scores for color, distortion and texture were 2.0 ± 0.9, 2.2 ± 0.9 and 2.3 ± 0.8, respectively. The mean pain score during treatment was 3.9 ± 0.8, and the mean duration of erythema was 30.7 ± 3.5 days. Only three patients developed hyperpigmentation at the treated site within a few months. Discussion: Ultra-pulse CO_2_ fractional laser MMP technique can effectively improve the condition of facial atrophic acne scars with limited adverse reactions.

## 1. Introduction

Acne vulgaris, a common inflammatory disease of the pilosebaceous unit, mainly attacks the young and middle-aged [1]. About 80.2% of the patients experience various sequelae after skin lesion remission, such as facial post-inflammatory erythema (PIE) or hyperpigmentation (PIH), and acne scars form up in around 43% of patients [2,3]. Acne scars can be roughly divided into atrophic scars and hypertrophic scars, with the former being more common [4]. Atrophic acne scars are prone to appear on the forehead, temples and bilateral cheeks [5]. Severe atrophic scars have a significant negative impact on the patient’s appearance, causing inferiority complex or even mental disorders [6].

According to their shapes, acne atrophic scars can be divided into icepick, boxcar and rolling, and, sometimes, the three types coexist. Accounting for the majority of acne scars (around 60–70%), icepick scars are characterized with narrow and deep epithelial tracts that extends to dermis or subcutaneous tissues. Boxcar scars are wider, round or oval-like depressions with sharp vertical edges. Rolling scars are the widest acne scars, with a diameter even up to 5 mm [4]. The undulant appearance is caused by the fibers anchored to the dermis, and these fibers hinder collagen reconstruction; thus, resurfacing them is the key to treatment success.

Atrophic acne scars can be treated with chemical peeling, surgical excision, dermabrasion, subcutaneous filling, fractional CO_2_ laser, fractional microneedle radiofrequency, etc. CO_2_ laser was widely applied in tissue engineering and clinical medicine [7]. Manstein D et al. proposed the “fractional photothermolysis” in 2004, which led to the widespread use of fractional lasers in the treatment of skin atrophic scars [8]. Fractional CO_2_ laser (10,600 nm), an effective and well-established technology, can flatten the edge of scars and promote the production of collagens in the depressions. As thermal energy is absorbed by the water in intracellular tissues, superficial skin is heated and vaporized [9]. The ultra-pulse CO_2_ laser has an impulse shorter than the time for thermal relaxation, thus minimizing the damage to normal tissue. Through fragmentizing the collagen matrix anchored to the subcutis and stimulating new collagen production, the fractional CO_2_ laser can effectively promote tissue remodeling and skin resurfacing [10]. However, the high thermal energy may bring with side effects, such as prolonged erythema, hypopigmentation, post-inflammatory hyperpigmentation and slow recovery [11]. 

The efficacy and safety of fractional CO_2_ laser for atrophic scars were much explored [12,13,14]. In 121 cases, we found that multiple sessions of CO_2_ fractional laser achieved a satisfactory effect in treating atrophic acne scars. The efficacy on rolling scars is better than that on boxcar and icepick scars [15]. In addition, a higher laser energy predicts a stronger effect, but a higher risk of persistent erythema and pigmentation [15]. These findings were similar to the findings of other studies [16]. Ultra-pulse CO_2_ fractional laser can penetrate by a maximum of 2–4 mm depth to the epidermis and upper papillary dermis [17]. Greater energy can realize a larger range of thermal damage, exerting a better therapeutic effect but more significant adverse reactions. Schweiger et al. reported that focal fractional laser treatment (FFLT), only focusing on atrophic scars other than normal skin tissues, could achieve an ideal effect and significantly reduce adverse reactions [18]. To reduce postoperative reactions, composite masks containing antimicrobial peptides and hyaluronic acid are recommended to promote skin wound repair after CO_2_ laser treatment [19]. Manual fractional technique (MFT) was used for hypertrophic scars and keloids in recent years, through which ultra-pulse CO_2_ laser is manually manipulated to drill into the scar, and the penetration depth can be controlled by the duration of laser energy release [20]. This technique was recently applied to atrophic scars as well [14]. However, it is difficult to achieve a desired effect after limited sessions of fractional CO_2_ laser alone, and so, a combination of multiple laser technologies, which are delivered in one session, should be designed to achieve more favorable outcomes. 

In this study, we performed CO_2_ laser with a new protocol integrating the above three techniques, named “multiple mode procedures (MMP)”, to treat acne atrophic scars. MMP contains three steps. Firstly, the focal atrophic scars were treated by focal fractional CO_2_ laser with a high density and energy. Secondly, manual fractional technique was performed to act on the edge of the hypertrophic scars. Thirdly, traditional fractional CO_2_ laser was performed at low density and energy on the scars and its surrounding skin. The efficacy and safety of MMP for atrophic acne scars were evaluated by the ECCA score, 4-Point Global Assessment Score (GAS), modified Manchester Scar Scale and the occurrence of adverse reactions. 

## 2. Methods

### 2.1. Clinical Data

In this retrospective cohort study, we collected clinical data from 103 patients with atrophic acne scars who received MMP in the Department of Cosmetic Dermatology of Nanjing Yijia Medical Aesthetic Clinic from December 2017 to January 2023. The study was approved by the Ethics Review Committee of Nanjing Yijia Medical Aesthetic Clinic (ethics number: 2022-001). Inclusion criteria: (1) two attending aesthetic physicians at the institution diagnosed the patient with atrophic acne scarring; (2) the patient received at least one session of MMP; (3) patients with complete clinical data and follow-up data. Exclusion criteria: patients who underwent other types of lasers, radiofrequency, surgery or chemical peeling before, during and within three months after treatment.

### 2.2. Treatment Procedures

#### 2.2.1. Preoperative Preparation

Facial condition was assessed preoperatively in all patients and the facial skin was cleaned. The facial photos were taken with one camera in the room. The pictures of the front, left (45° and 90° angles) and right (45° and 90° angles) face were obtained and archived. An informed consent form was signed by every participant. The plastic wrap covered with compound lidocaine cream (2.5% lidocaine and 2.5% proprivacaine, 50 mg, produced by Beijing Ziguang Pharmaceutical Co., Ltd., Beijing, China) was applied for one hour to achieve facial surface anesthesia. In this study, an ultra-pulse CO_2_ fractional lasers apparatus (Fimilift, Alma^TM^ Lasers, Caesarea, Israel) was used with pixel and F100 handpieces.

#### 2.2.2. Details of MMP Technique

Parameters and procedures of MMP: after 60 min of surface anesthesia, the skin was routinely cleansed and disinfected. Laser operation was divided into three steps:

##### FFLT

Fractional CO_2_ laser, manipulated by pixel 9 × 9 handpiece in an aesthetic mode, was restricted on the atrophic acne scar surface and the surrounding skin surface. The laser energy was adjusted according to the shape and area of the atrophic scar, mainly aimed at the bottom of the scar. The relatively high laser energy density was used in FFLT (80–100 mJ/pixel).

##### MFT

The atrophic scar was stretched by the operator’s fingers as much as possible, so as to flatten the atrophic scar depression till a hypertrophic scar-like appearance rose around the scar. Then, the CO_2_ laser was applied to the hypertrophic scar-like bulges in the form of MFT with F100 handpiece in the surgical mode. The interval between two points was generally 2–4 mm and the depth of laser penetration was adjusted according to the improvement of these peripheral bulges. 

##### Fractional Laser

The pixel handpiece in the aesthetic mode was used with a low density (7 × 7) and relative lower energy (20–40 mJ/pixel) to widely scan the facial skin. The energy was adjusted according to the age, type, location and severity of acne scars and the skin texture.

**Figure 1:**
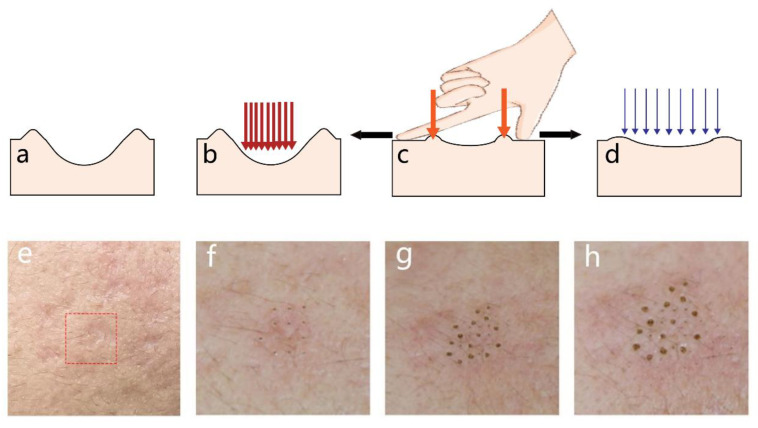
Schematic diagram and clinical photos of MMP. (**a**,**e**): A cross-sectional diagram and a clinical photo of acne atrophic scars (in the dashed box) before treatment. (**b**,**f**) FFLT step: Apply high-energy, high-density fractional laser (red arrows) to the bottom and periphery of the scar. (**c**,**g**) MFT step: Use the finger to stretch the scar so that the periphery of the scar forms a slight bulges; then apply an artificial laser (orange arrows) drilling to the edge of the scar, until the peripheral bulges become flat. (**d**,**h**) Fractional CO_2_ laser: Use relatively low-energy, low-density fractional CO_2_ laser (blue arrows) to scan the whole treatment aera. Abbreviation: MMP: Multiple Mode Procedures; FFLT: focal fractional laser treatment; MFT: manual fractional technique.

**Figure 2:**
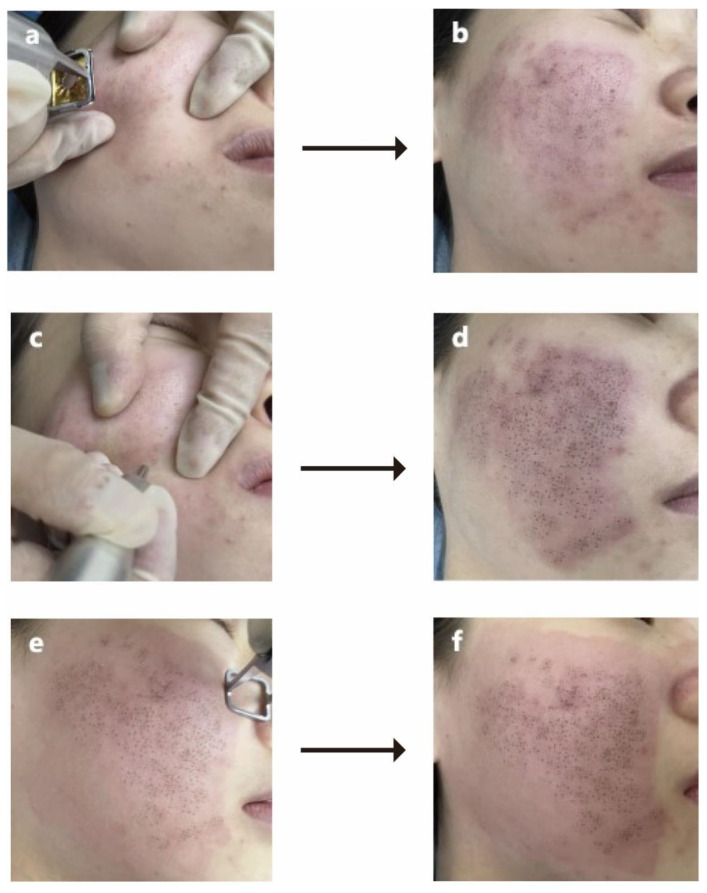
Clinical diagram of MMP. (**a**,**b**) Before and after the FFLT step; (**c**,**d**) before and after treatment with MFT step; (**e**,**f**) before and after fractional CO_2_ laser scanning. Abbreviation: MMP: Multiple Mode Procedures; FFLT: focal fractional laser treatment; MFT: manual fractional technique.

A schematic diagram (Figure 1) details the procedures and mechanisms of MMP with intuitive clinical photos. The operation methods and instant responses to each procedure of MMP are shown in Figure 2. After the above procedures, the patients received postoperative care. 

#### 2.2.3. Postoperative Care

Immediately after treatment, growth factor solution (Beifuji, Zhuhai Yiyao Biopharmaceutical Co., Ltd.) was sprayed on the treated face area. The patient was instructed to spray the growth factor solution on the wound surface (two or three times a day, for two weeks) until the scab completely fell off. Then, ice bag and/or cold compress (Aikangda, Jiangsu Yisheng Biotechnology Co., Ltd.) was used to relieve redness and pain, until the discomfort was well controlled. During the first three days after treatment, halomethasone triclosan was creamed twice a day on the laser-treated site. The patient was advised to avoid sunlight as much as possible for one month after treatment. Physical sun protection was recommended in the outside, and the photosensitive food should be avoided. Generally, it is recommended that the interval between two rounds of treatment should be at least three months. All patients were followed for at least three months after the last treatment. 

### 2.3. Assessment of Clinical Characteristics

The following data were extracted from the medical records: (1) age at the first treatment; (2) gender; (3) scar types: icepick, boxcar and rolling scars (because most patients had multiple types of acne atrophic scars, we chose the type that accounted for at least 50% of all scars as the main acne scar type for statistics.); (4) duration of atrophic scars; (5) the number of treatment sessions; (6) ECCA score [21]: consisting of 6 items designed to easily and quickly assess the severity of acne scars by a global score; (7) 4-point global assessment score: evaluated before and after treatment; 0–4 points were given based on the description of facial acne scar (0, no improvement [0–25%]; 1, light improvement [26–50%]; 2: significant improvement [51–75%]; 3: obvious improvement [75–100%]); (7) score of modified Manchester Scar Scale to assess the changes in the color, distortion and texture of the scar lesions [22]; 1 to 4 was given to each item and the total score was obtained by adding scores of all items (color: perfect—1, slight mismatch—2, obvious mismatch—3; gross mismatch—4; distortion: none—1, mild—2, moderate—3, severe—4; texture: normal—1, just palpable—2, firm—3, hard—4); (8) adverse reactions: visual analogue score [23] to assess the degree of pain during treatment (a higher score represented a more serious pain), postoperative pigmentation (lasting more than one month), duration of persistent erythema, secondary infection, acneiform eruption and scar deterioration. The post-treatment evaluation was performed by two dermatologists who were unaware of the study. The postoperative symptoms were reported by the participants and confirmed by dermatologists during the follow-up visits.

### 2.4. Statistic Analysis

All statistical analyses were run by R software (version 3.5.2). Normally distributed continuous data and skewedly distributed continuous data were expressed as mean ± standard deviation and median (min-maximum), respectively. Categorical variables were displayed as proportion (%). Differences between variables were verified by chi-square test (categorical variables), *t*-test or Mann–Whitney U test (continuous variables). *p* < 0.05 was considered statistically significant.

## 3. Results

All 103 patients received one to four sessions of treatment and completed the follow-up (Table 1). Among them, 38 patients received one session of treatment, 50 patients received two sessions, 12 patients received three sessions and 3 patients received four sessions. The clinical features of patients with atrophic scars are shown in Table 1. Included were 20 males (19.4%) and 83 females (80.6%), with an age of 28.6 ± 5.1 years. The average duration of the acne scars was 10.1 (min to max: 2–40) years. There were 49 (47.6%) patients with a main type of boxcar scars, 35 (34.0%) with icepick scars and 19 (18.4%) with rolling scars. Among the 103 patients who received laser therapy, 14 patients still had mild active acne lesions on the face during treatment, and 6 patients only intermittently used some acne topical medication, such as Benzoyl peroxide gel, Fusidic acid cream or Clindamycin gel, to control the scars.

**Figure 3:**
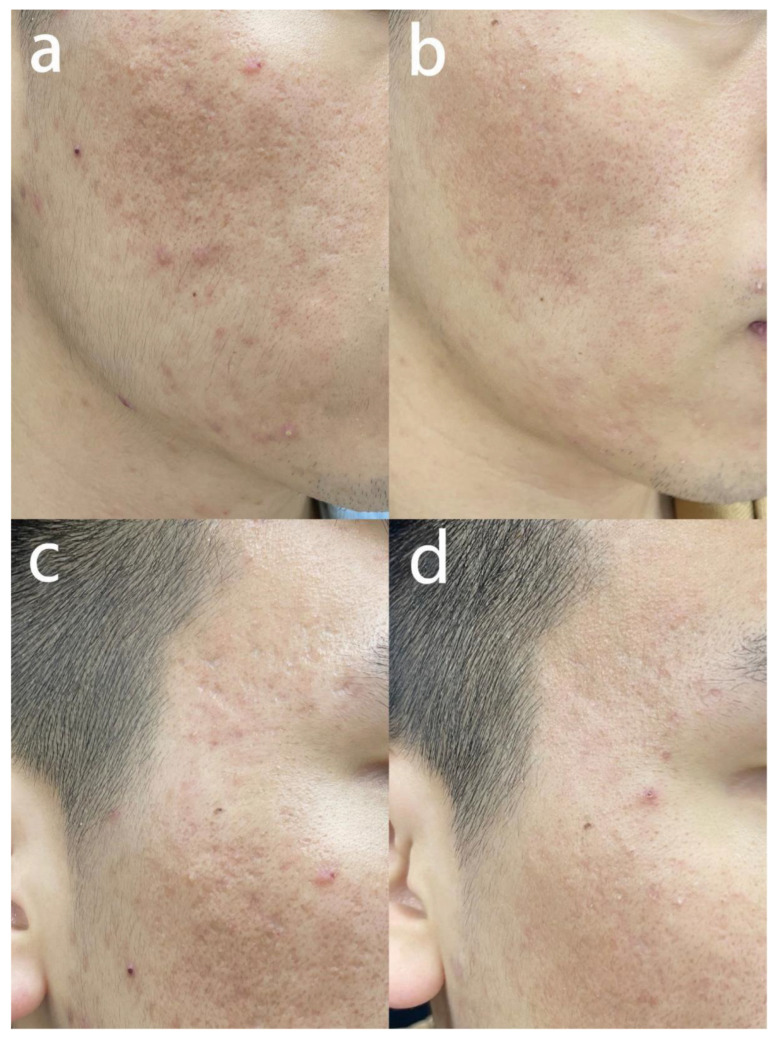
Fractional CO_2_ laser of MMP for treating rolling scars. (**a**,**c**) The patient was a 25-year-old male with an ECCA score of 330 before treatment. (**b**,**d**) Three months after the second session, ECCA score decreased to 150; the scores for color, distortion and texture were 3, 2 and 3, respectively; GAS score was 3. Abbreviation: MMP: Multiple Mode Procedures; ECCA: échelle d’évaluation clinique des cicatrices d’acné.

**Figure 4:**
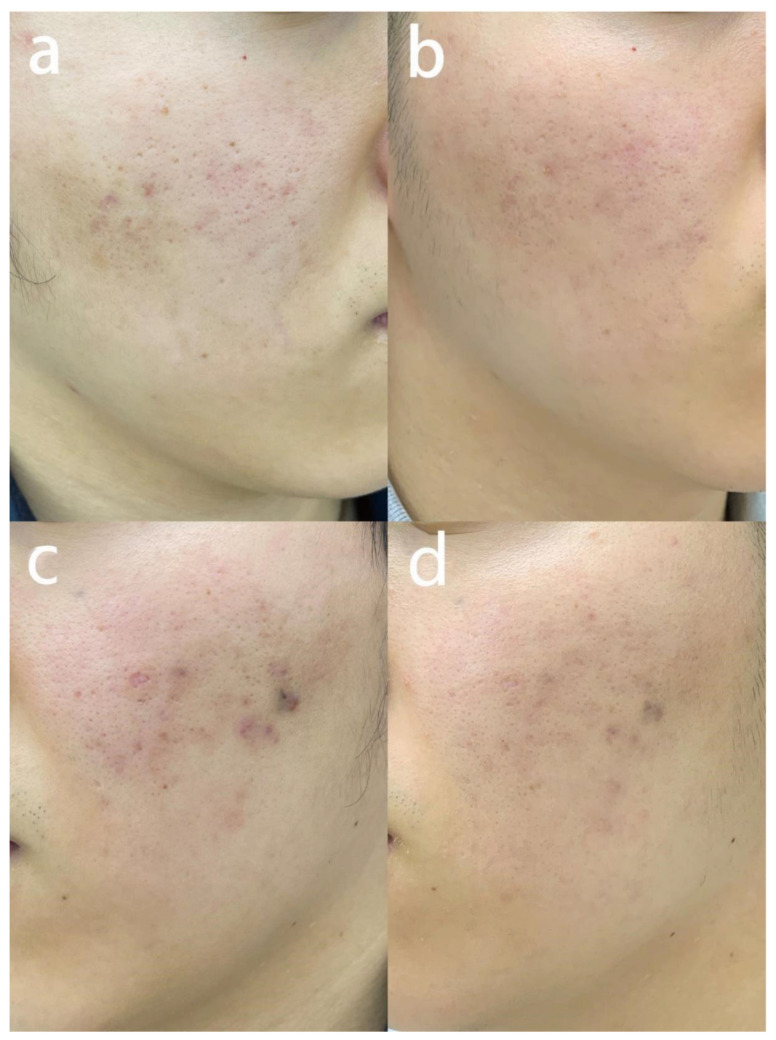
Fractional CO_2_ laser of MMP for treating icepick scars. (**a**,**c**) The patient was a 25-year-old male with an ECCA score of 180 before treatment. (**b**,**d**) Three months after the first treatment, ECCA score was 85; the scores for color, distortion and texture were 3, 3 and 3, respectively; GAS score was 3. Abbreviation: MMP: Multiple Mode Procedures; ECCA: échelle d’évaluation clinique des cicatrices d’acné.

**Figure 5:**
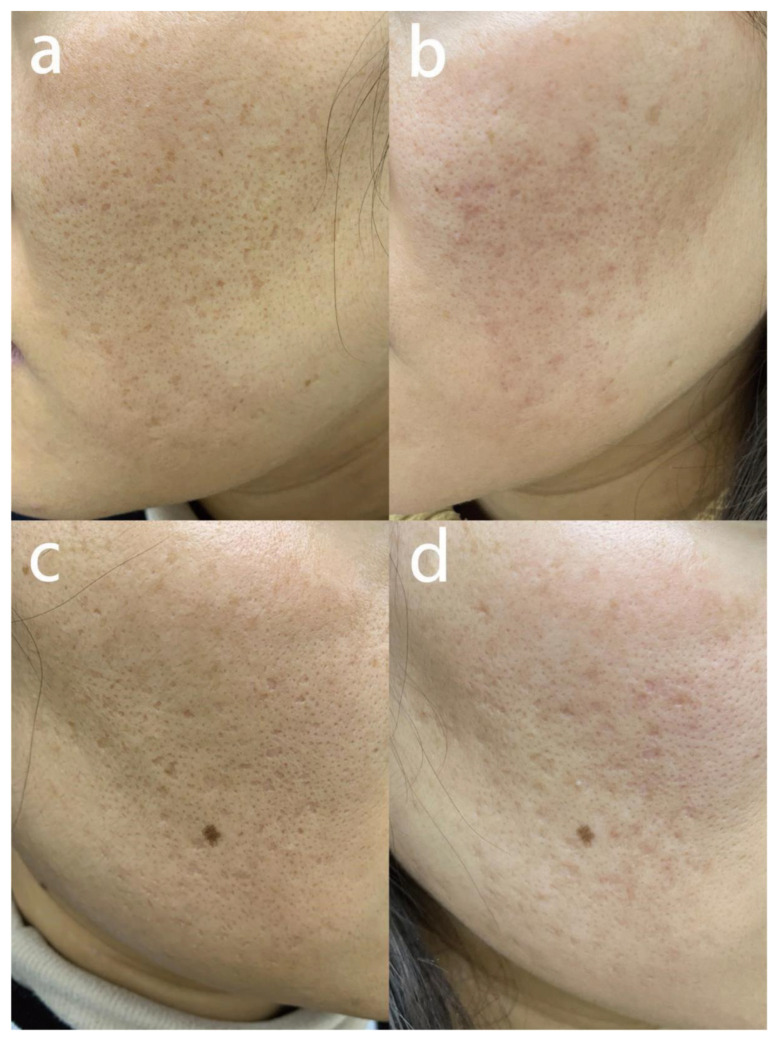
Fractional CO_2_ laser of MMP for treating boxcar and icepick scars. (**a**,**c**) The patient was a 31-year-old female with an ECCA score of 125 before treatment. (**b**,**d**) Three months after the first treatment, ECCA score was 85; the scores for color, distortion and texture were 3, 3 and 3, respectively; GAS score was 3. Abbreviation: MMP: Multiple Mode Procedures; ECCA: échelle d’évaluation clinique des cicatrices d’acné.

All types of atrophic acne scars showed positive changes, and representative photos are shown in Figure 3, Figure 4 and Figure 5. Therapeutic effect of the 103 atrophic acne scar patients is displayed in Table 2. Overall, the ECCA score of all patients decreased from 162.7 ± 47.6 to 93.1 ± 34.7 after MMP treatment, with a statistically significant difference (*p* < 0.001). The average GAS score was 2.3. The average scores for improvements in color, unevenness and texture were 2.0 ± 0.9, 2.2 ± 0.9, 2.3 ± 0.8, respectively, with the highest in texture. Comparing the improvement scores of three different types of scars, MMP exerted the strongest effect on boxcar scars (GAS, 2.7 ± 0.8; color, 2.4 ± 0.9; distortion, 2.5 ± 0.9; texture, 2.6 ± 0.8), followed by icepick scars (GAS, 1.67 ± 0.8; color, 1.5 ± 0.7; deformation, 1.8 ± 0.8; texture, 1.87 ± 0.8) and rolling scars (GAS, 2.3 ± 0.8; color, 1.8 ± 0.6; deformation, 2.1 ± 0.8; texture, 2.3 ± 0.7) (Table 3).

All patients tolerated the procedures well and showed varying degrees of burning, pain, edema and erythema, all significantly relieved after cold compress. The mean pain score was 3.9 ± 0.8 during the treatment. and the average duration of erythema was 30.7 ± 3.5 days. Three patients developed pigmentation, which was resolved within a 3-month treatment with one–two intense pulsed light therapy combined with anti-pigmentation cream. No patients exhibited skin infection, acneiform eruption and/or worsening scarring.

## 4. Discussion

In using fractional CO_2_ laser for treating atrophic acne scars, CO_2_ laser is uniformly split into evenly arranged fractional spots, which target water chromophore in the skin tissue to exert microthermal damage, thus triggering repair response, contraction and regeneration of dermal collagens in the surrounding normal skin tissue. However, due to its limited penetration depth, conventional fractional CO_2_ laser can only achieve satisfactory results for many atrophic scars after multiple (usually more than five) sessions [24]. As a consequence, patients are often prone to persistent pigmentation, erythema, skin sensitivity and other adverse reactions [11]. Because the depth of an atrophic acne scar varies, extensive treatment of the whole layer of scar is often needed. Fractional CO_2_ laser can only penetrate into the scar by a depth of 2–4 mm at most [9]. In contrast, laser drilling using MFT technology can convey the energy of CO_2_ laser to deeper scar tissues. In addition, the superficial skin, the texture of scar and its surrounding skin should also be simultaneously treated to achieve a high satisfaction in patients. In the present study, we proved that a combination of conventional fractional CO_2_ laser, MFT and FFLT (referred as MMP technique) was effective and safe to treat facial acne atrophic scars.

As the first procedure in MMP, FFLT can be used to focally treat atrophic acne scars with high energy, without treating the normal tissue surrounding the scars. Fabbrocini G et al. reported that the atrophic acne scars were treated with high-concentration trichloroacetic acid (TCA) solution focally, every 4 weeks for a total of three sessions [25]; the clinical examination showed favorable changes in the depth and appearance of the skin scar [25]. They called this technology the “chemical reconstruction of skin scar” (CROSS) method. The key in this method was to treat acne scars with strong peeling acid, and focus it only on scars; meanwhile, the surrounding normal tissue was protected to reduce the probability of large scale hyperpigmentation and erythema [25]. Schweiger et al. used the same FFLT technique to treat atrophic acne scars, achieving satisfactory outcomes in all the six patients [26]. Similar to the CROSS method, FFFT poses high-energy laser only on the atrophic acne scars, thereby damaging deeper scar tissues, stimulating the tissue regeneration and flattening the depression at the bottom of the scar. In traditional fractional laser therapy, a comprehensive plain scanning is often performed due to the large treatment area, thus resulting in an extensive wound and unexpected adverse reactions [11]. In order to ensure the safety of treatment, the operator usually sets low energy parameters, which may discount the therapeutic effect. Compared with the traditional plain scanning, the focal energy in FFLT has a stronger ability to fragment scar tissues and stimulate collagen synthesis [18,27]. Although post-laser reactions may occur in a short term, FFLT is much preferred by patients, because it brings smaller wounds and milder pain. In the present study, the FFLT in MMP caused local skin edema in a short time, making the tissue around the atrophic scar appear slightly hypertrophic. If the scar was stretched by fingers, the bulges around became more obvious.

The second procedure in MMP is MFT to grind the bulges around the scar, especially when the atrophic scar is unfolded by fingers. Tan J et al. proposed that MFT combined with fractional CO_2_ laser can damage deep scar tissues, which is highly therapeutic for hypertrophic scars [20]. Unlike traditional large-area mechanical or laser grinding, MFT can accurately and effectively grind the uneven part around the scar. Compared with traditional laser ablation, spot “drilling” brings a smaller wound, which reduces adverse reactions. In another study, MFT was used to treat the base rather than the periphery of trophic scars; the results showed that CO_2_ laser photothermal effect could “flatten” the depressed scar [18]. In addition, in the early stage of MFT, patients may observe the temporary deepening of the scar, which causes anxiety. Tn this study, we used MFT method to treat the bulges around the scar, which avoided temporary deepening of the atrophic scars. In the final procedure of MMP, we used the traditional fractional lase, but with a relatively low laser energy and a scanning density, to extensively treat the scar and the skin around. Both procedures effectively reduced fine skin scars and pores, softened skin texture.

In the present study, the ECCA score of the patients decreased from 162.7 ± 47.6 to 93.1 ± 34.7, with an improvement rate of 42.8%. This was only the effect obtained within one–four sessions. The decline of ECCA score in this study was comparable to those achieved by multiple sessions of traditional fractional CO_2_ laser in other studies. Jin W et al. evaluated the efficacy of manual fractional technique, finding that the mean ECCA score reduced from 67.50 ± 23.98 to 45.68 ± 18.57 (an improvement less than that achieved by MMP). Additionally, Wang Y et al. observed that the ECCA score decreased from 89.12  ±  7.08 to 69.21  ±  3.15 after treatment with ultrapulse CO_2_ fractional laser alone [12], displaying an efficacy weaker than that in this study. In another study, by Kang WH et al., a combination of dot chemical peeling, subcision and several sessions of fractional CO_2_ laser harvested ideal results (GAS score: 2.0 ± 0.7), but these results could only appear after at least 4–6 months of treatment [13]. Compared with other lasers, such as the Nd: YAG and pulsed dye laser (PDL), fractional CO_2_ laser also performs better in treating acne scars. Ali et al. compared the efficacy of fractional CO_2_ laser and Q-switched 1064 nm Nd: YAG in sixty-four patients with severe acne scars, and found that the fractional CO_2_ laser achieved a more evident improvement [28]. Another study investigated the efficacy of fractional CO_2_ laser and the PDL on surgical scars. The results displayed that fractional CO_2_ laser was more powerful in improving the pliability and thickness [29].

Interestingly, of the three types of atrophic scars, the boxcar scars responded to MMP most and the icepick scars least obviously. In previous research, we found that rolling scars presented the strongest response after conventional fractional CO_2_ laser treatment [15]. MMP can effectively compensate for the weak effect of traditional mode on boxcar scars. For icepick scars, fractional laser alone fails to reach a satisfactory efficacy, and its combination with CROSS technique or surgical excision should be considered.

All features of atrophic scars, including color, distortion and texture, demonstrated encouraging changes at three months after treatment. The modified Manchester Scar Scale showed that, after treatment with MMP, the improvement score in color was the least, with an average of 2.0 ± 0.9, while those of deformation and texture were 2.2 ± 0.9 and 2.3 ± 0.8, respectively. We believe that this may be due to the multiple laser interventions in MMP that provide a higher laser energy than the traditional model, as well as the post-treatment care. However, in this study, we found that the persisting erythema subsided within about one month after treatment in most patients, and only three patients developed persistent hyperpigmentation, which was significantly improved within three months after intensive pulse light combined with anti-pigmentation cream. We believe that these mild adverse reactions were mainly related to growth factor solution sprayed on laser wounds [30]. In addition, halomethasone cream containing antibiotic ingredients can also reduce excessive inflammatory reaction after laser, and significantly prevent postoperative pigmentation and secondary infection. Educating patients to take sunscreen is an effective measure to avoid postoperative erythema and pigmentation.

This study also had some limitations. First, this was a single-center, small-sample retrospective study, and randomized multi-center clinical trials will be helpful to validate the findings in this study. Second, a control group was not set up. Third, this study only used one fractional laser instrument and it is unknown whether the results in this study could be generalized into other fractional CO_2_ laser therapies. Fourth, this study did not validate the effectiveness of MMP technology in treating atrophic scars from a histopathological perspective.

## 5. Conclusions

As a new modality, MMP can bring a good clinical efficacy and few adverse reactions in the treatment of atrophic acne scars.

## Figures and Tables

**Table 1:** Clinical characteristics of the 103 atrophic acne scar patients.

	Mean ± SD
Age (Years)	28.6 ± 5.1
ECCA scores before treatment	162.7 ± 47.6
Duration (Years)	10.1 ± 5.1
	N (%)
Gender	
Female	83 (80.6%)
Male	20 (19.4%)
Scar type	
Boxcar	49 (47.6%)
Icepick	35 (34.0%)
Rolling	19 (18.4%)
Accompanied by active acne lesions	
Yes	14 (13.6%)
No	89 (86.4%)

**Table 2:** Therapeutic effect of the 103 atrophic acne scar patients.

	Mean ± SD
The number of treatment sessions	1.8 ± 0.8
GAS score	2.3 ± 0.9
ECCA score after treatment	93.1 ± 34.7
Modified Manchester Scar Scale (Color)	2.0 ± 0.9
Modified Manchester Scar Scale (Distortion)	2.2 ± 0.9
Modified Manchester Scar Scale (Texture)	2.3 ± 0.8
Pain level	3.9 ± 0.8
Persistent erythema (Days)	30.7 ± 3.5
Pigmentation (one month after treatment)	
No	100 (97.1%)
Yes	3 (2.9%)

**Table 3:** Therapeutic effects of MMP for different types of acne atrophic scars.

Scar Type	Boxcar	Icepick	Rolling	*p*-Value
Number	49	35	19	
ECCA score before treatment	165.5 ± 35.9	143.4 ± 53.6	190.8 ± 48.9	<0.001
ECCA score after treatment	83.3 ± 29.6	92.6 ± 33.9	119.5 ± 36.1	<0.001
Modified Manchester Scar Scale (Color)	2.4 ± 0.9	1.5 ± 0.7	1.8 ± 0.6	<0.001
Modified Manchester Scar Scale (Distortion)	2.5 ± 0.9	1.8 ± 0.8	2.1 ± 0.8	0.002
Modified Manchester Scar Scale (Texture)	2.6 ± 0.8	1.7 ± 0.8	2.3 ± 0.7	<0.001
GAS Score	2.7 ± 0.8	1.7 ± 0.8	2.3 ± 0.8	<0.001

## Data Availability

The data presented in this study are available on request from the corresponding author. The data are not publicly available due to private issues of all subjects.

## References

[1] Lynn D.D., Umari T., Dunnick C.A., Dellavalle R.P. (2016). The epidemiology of acne vulgaris in late adolescence. Adolesc. Health Med. Ther..

[2] Thiboutot D.M., Dréno B., Abanmi A., Alexis A.F., Araviiskaia E., Barona Cabal M.I., Bettoli V., Casintahan F., Chow S., da Costa A. (2018). Practical management of acne for clinicians: An international consensus from the Global Alliance to Improve Outcomes in Acne. J. Am. Acad. Dermatol..

[3] Abad-Casintahan F., Chow S.K., Goh C.L., Kubba R., Hayashi N., Noppakun N., See J., Suh D.H., Xiang L.H., Kang S. (2016). Frequency and characteristics of acne-related post-inflammatory hyperpigmentation. J. Dermatol..

[4] Connolly D., Vu H.L., Mariwalla K., Saedi N. (2017). Acne Scarring-Pathogenesis, Evaluation, and Treatment Options. J. Clin. Aesthetic Dermatol..

[5] Clark A.K., Saric S., Sivamani R.K. (2018). Acne Scars: How Do We Grade Them?. Am. J. Clin. Dermatol..

[6] Samuels D.V., Rosenthal R., Lin R., Chaudhari S., Natsuaki M.N. (2020). Acne vulgaris and risk of depression and anxiety: A meta-analytic review. J. Am. Acad. Dermatol..

[7] Khorasani M., Gibson I., Ghasemi A.H., Hadavi E., Rolfe B. (2023). Laser subtractive and laser powder bed fusion of metals: Review of process and production features. Rapid Prototyp. J..

[8] Manstein D., Herron G.S., Sink R.K., Tanner H., Anderson R.R. (2004). Fractional photothermolysis: A new concept for cutaneous remodeling using microscopic patterns of thermal injury. Lasers Surg. Med..

[9] Sobanko J.F., Alster T.S. (2012). Management of acne scarring, part I: A comparative review of laser surgical approaches. Am. J. Clin. Dermatol..

[10] Prignano F., Campolmi P., Bonan P., Ricceri F., Cannarozzo G., Troiano M., Lotti T. (2009). Fractional CO_2_ laser: A novel therapeutic device upon photobiomodulation of tissue remodeling and cytokine pathway of tissue repair. Dermatol. Ther..

[11] Prado A., Andrades P., Danilla S., Benitez S., Reyes S., Valenzuela G., Guridi R., Fuentes P. (2008). Full-face carbon dioxide laser resurfacing: A 10-year follow-up descriptive study. Plast. Reconstr. Surg..

[12] Wang Y., Yu W., Zhang J., Li J. (2022). Effect and Safety Analysis of PRP and Yifu Combined with Ultrapulsed CO_2_ Lattice Laser in Patients with Sunken Acne Scar. J. Healthc. Eng..

[13] Kang W.H., Kim Y.J., Pyo W.S., Park S.J., Kim J.H. (2009). Atrophic acne scar treatment using triple combination therapy: Dot peeling, subcision and fractional laser. J. Cosmet. Laser Ther..

[14] Jin W., Li Z., Jin Z., Jin C. (2020). A novel technique for treating atrophic facial scars in Asians using ultra-pulse CO_2_ laser. J. Cosmet. Dermatol..

[15] Li B., Ren K., Yin X., She H., Liu H., Zhou B. (2022). Efficacy and adverse reactions of fractional CO_2_ laser for atrophic acne scars and related clinical factors: A retrospective study on 121 patients. J. Cosmet. Dermatol..

[16] Fang F., Yang H., Liu X., Ding H., Yang Y., Ge Y., Lin T. (2022). Treatment of acne scars with fractional carbon dioxide laser in Asians: A retrospective study to search for predicting factors associated with efficacy. Lasers Med. Sci..

[17] Preissig J., Hamilton K., Markus R. (2012). Current Laser Resurfacing Technologies: A Review that Delves Beneath the Surface. Semin. Plast. Surg..

[18] Hongjin W., Bingrong Z., Shufen X., Jiaan Z., Jin L., Juan L., Fei Y., Shen W., Lichao Z., Dan L. (2015). The efficacy and adverse reactions of fractional CO_2_ laser for treatment of atrophic acne scars. Chin. J. Dermatol..

[19] Zhang J.A., Liu J., Wu H.J., Xu Y., Si C.C., Zhou B.R., Luo D. (2019). The effects of Antimicrobial Peptides and Hyaluronic Acid compound mask on wound healing after ablative fractional Carbon Dioxide laser resurfacing. J. Cosmet. Laser Ther..

[20] Lei Y., Li S.F., Yu Y.L., Tan J., Gold M.H. (2017). Clinical efficacy of utilizing Ultrapulse CO_2_ combined with fractional CO_2_ laser for the treatment of hypertrophic scars in Asians-A prospective clinical evaluation. J. Cosmet. Dermatol..

[21] Dreno B., Khammari A., Orain N., Noray C., Mérial-Kieny C., Méry S., Nocera T. (2007). ECCA grading scale: An original validated acne scar grading scale for clinical practice in dermatology. Dermatology.

[22] Beausang E., Floyd H., Dunn K.W., Orton C.I., Ferguson M.W. (1998). A new quantitative scale for clinical scar assessment. Plast. Reconstr. Surg..

[23] Delgado D.A., Lambert B.S., Boutris N., McCulloch P.C., Robbins A.B., Moreno M.R., Harris J.D. (2018). Validation of Digital Visual Analog Scale Pain Scoring With a Traditional Paper-based Visual Analog Scale in Adults. J. Am. Acad. Orthop. Surg. Glob. Res. Rev..

[24] Zhang D.D., Zhao W.Y., Fang Q.Q., Wang Z.C., Wang X.F., Zhang M.X., Hu Y.Y., Zheng B., Tan W.Q. (2021). The efficacy of fractional CO_2_ laser in acne scar treatment: A meta-analysis. Dermatol. Ther..

[25] Fabbrocini G., Cacciapuoti S., Fardella N., Pastore F., Monfrecola G. (2008). CROSS technique: Chemical reconstruction of skin scars method. Dermatol. Ther..

[26] Schweiger E.S., Sundick L. (2013). Focal Acne Scar Treatment (FAST), a new approach to atrophic acne scars: A case series. J. Drugs Dermatol..

[27] Qin L., Kaiping Z., Jianbo W., Yuehua Y., Guifeng X., Jiquan S. (2019). The efficacy and security of focal fractional laser in treatment of atrophic acne scars. Chin. J. Med. Aesthet. Cosmetol..

[28] Asilian A., Salimi E., Faghihi G., Dehghani F., Tajmirriahi N., Hosseini S.M. (2011). Comparison of Q-Switched 1064-nm Nd: YAG laser and fractional CO_2_ laser efficacies on improvement of atrophic facial acne scar. J. Res. Med. Sci..

[29] Kim D.H., Ryu H.J., Choi J.E., Ahn H.H., Kye Y.C., Seo S.H. (2014). A comparison of the scar prevention effect between carbon dioxide fractional laser and pulsed dye laser in surgical scars. Dermatol. Surg..

[30] Lee C.J., Whang J.H., Lazova R., Ciesielski T.E., Thomson J.G., McCarthy T., Persing J.A. (2007). Growth factor expression with different wound treatments after laser resurfacing. Aesthetic Surg. J..

